# Assessing the properties of patient-specific treatment effect estimates from causal forest algorithms under essential heterogeneity

**DOI:** 10.1186/s12874-024-02187-5

**Published:** 2024-03-13

**Authors:** John M. Brooks, Cole G. Chapman, Brian K. Chen, Sarah B. Floyd, Neset Hikmet

**Affiliations:** 1 Center for Effectiveness Research in Orthopaedics - Arnold School of Public Health Greenville, 915 Greene Street #302D, Columbia, SC 29208-0001 USA; 2 University of South Carolina Arnold School of Public Health, Health Services Policy & Management, Columbia, SC USA; 3 Department of Pharmacy Practice and Science Iowa City, University of Iowa, Iowa, USA; 4 Center for Effectiveness Research in Orthopaedics, Greenville, SC USA; 5 Clemson University College of Behavioral Social and Health Sciences, Public Health Sciences, Clemson, South Carolina USA; 6 Department of Integrated Information Technology, Innovation Think Tank Lab @ USC, University of South Carolina College of Engineering and Computing, Columbia, SC USA

**Keywords:** Machine learning, Causal Forest Algorithm (CFA), Treatment effect estimation, Simulation modeling, Linear probability estimators

## Abstract

**Background:**

Treatment variation from observational data has been used to estimate patient-specific treatment effects. *Causal Forest Algorithms (CFAs)* developed for this task have unknown properties when treatment effect heterogeneity from unmeasured patient factors influences treatment choice – *essential heterogeneity*.

**Methods:**

We simulated eleven populations with identical treatment effect distributions based on patient factors. The populations varied in the extent that treatment effect heterogeneity influenced treatment choice. We used the generalized random forest application (CFA-GRF) to estimate patient-specific treatment effects for each population. Average differences between true and estimated effects for patient subsets were evaluated.

**Results:**

CFA-GRF performed well across the population when treatment effect heterogeneity did not influence treatment choice. Under essential heterogeneity, however, CFA-GRF yielded treatment effect estimates that reflected true treatment effects only for treated patients and were on average greater than true treatment effects for untreated patients.

**Conclusions:**

Patient-specific estimates produced by CFAs are sensitive to *why* patients in real-world practice make different treatment choices. Researchers using CFAs should develop conceptual frameworks of treatment choice *prior to estimation* to guide estimate interpretation *ex post*.

**Supplementary Information:**

The online version contains supplementary material available at 10.1186/s12874-024-02187-5.

## Introduction

Developing *patient-specific treatment effect evidence* to guide individualized treatment decision-making is a cornerstone of patient-centered care [1–3]. The need for patient-specific evidence follows from the acknowledged breadth of outcome variation across patients receiving the same treatment. [4–10]. This phenomenon is known as *treatment effect heterogeneity* and is defined as “nonrandom variation in the direction of magnitude of a treatment effect” [11]. With their restrictive inclusion/exclusion criteria, randomized controlled trials cannot generate appropriate patient-specific evidence for many patients [4, 11–14]. As an alternative, observational data provide treatment variation within the context of real-world practice and a diversity of patients well beyond those evaluated in RCTs [2, 3, 12, 15, 16]. The traditional approach to estimate patient-specific treatment effects using observational data is to use parametric estimators and assign to each patient an estimated treatment effect from a “reference class” of patients [17–22]. Reference classes are defined a priori by the researcher based on combinations of measured patient factors that are conceptually associated with treatment effect heterogeneity [17–22]. The need to specify reference classes a priori has been described as “the central problem when using group evidence to forecast outcomes (or treatment effects) in individuals” [18]. Even with a small number of measured patient factors, a patient could be placed in many reference classes, leaving it unclear which class is best aligned to the patient [10, 17, 18].

Causal forest algorithms (CFAs) have been proposed to estimate patient-specific treatment effects in a manner that essentially assigns patients to reference classes *ex post* using information from the data, thereby eliminating the need to assign patients to reference classes a priori [23–33]. Simulation modeling has shown that CFAs can accurately estimate patient-specific treatment effects in scenarios in which treatment effect heterogeneity does not influence treatment choice [24, 26–29, 34–37]. However, in many real-world scenarios it is conceivable that unmeasured patient factors associated with treatment effectiveness influence treatment choice. This is called *essential heterogeneity* or sorting on the gain in the econometrics literature [38–51]. The properties of parametric treatment effect estimators under essential heterogeneity are well known [38–51]. However, the impact of essential heterogeneity on patient-specific treatment effect estimates using CFAs has not been evaluated. In this paper, we contrast the properties of patient-specific treatment effect estimates using the causal forest algorithm within the generalized random forests application (CFA-GRF) across simulation scenarios that vary in the extent that unmeasured patient factors associated with treatment effectiveness influence treatment choice.

### Methodological background

Assigning patients into appropriate reference classes using observational data either a priori with parametric estimators or *ex post* through a CFA does not ensure that the resulting treatment effect estimates are appropriate for each patient. The conventional criticism of using observational data to estimate treatment effects is the risk of omitted variable bias in which unmeasured factors with direct effects on study outcomes are distributed differently between treated and untreated patients [52]. However, even if patients were assigned to appropriate reference classes and omitted variable bias risk is mitigated through study design, a single treatment effect estimate for a reference class may not be appropriate for each patient within a class. The econometric literature has shown that parametric estimators yield average treatment effect estimates for patient subsets based on treatment choice [38–67]. Under the assumption of no omitted variable bias, regression-based estimators yield unbiased estimates of the average treatment effect for the subset patients who chose treatment or the *average treatment effect on the treated* (ATT) [43, 48–50, 54, 57, 60, 68, 69]. Consequently, if treatment choice in an empirical setting was influenced by unmeasured patient factors related to treatment effectiveness – *essential heterogeneity* – the parametric estimate of ATT for a reference class will overstate the true treatment effects for the untreated patients in the class [39, 49, 50, 70]. Researchers using parametric estimators have learned not to generalize a single parametric treatment effect estimate to all patients in a population [38, 43, 47–51, 53, 55, 56, 58, 59, 61, 67, 70, 71].

In contrast, the properties of estimated patient-specific treatment effects from CFAs under essential heterogeneity have not been explored. Simulation research has demonstrated that CFAs accurately yield patient-specific treatment effects under the broad condition of *ignorability* [24, 26–29, 34–36]. Ignorability assumes that omitted variable bias does not exist within an empirical setting. However, ignorability also assumes that essential heterogeneity does not exist. These dual assumptions can be described using potential outcome notation. Define Y_1i_ and Y_0i_ as the potential outcomes for patient “i” when treated and untreated, respectively, and (Y_1i_ – Y_0i_) is the true potential treatment effect for patient “i”. Define T_i_ as the observed treatment choice for patient “i” and X_i_ as the set of measured patient factors available to the researcher. Ignorability is broadly defined as (Y_1i_, Y_0i_) $\perp$ T_i_ | X_i_ or conditional on X_i_, treatment choice is independent of *both* potential patient outcomes [72]. As such, ignorability implies the following two distinct assumptions.

1.1 $$\left({{\text{Y}}}_{0{\text{i}}}\right) \perp {{\text{T}}}_{{\text{i}}} \left|{{\text{X}}}_{{\text{i}}}\right.$$

Assumption (I.1) says that, within a reference class of patients based on X_i_, treatment choice is unrelated to *untreated potential outcomes* across patients. Or stated differently, treatment choice is unrelated to unmeasured patient factors associated with Y_0i_. Assuming (I.1) eliminates the risk of omitted variable bias in an observational study [52].

Even if assumption (I.1) is true though, treatment effects may remain heterogeneous within a reference class defined by X_i_. With respect to this heterogeneity, ignorability further assumes:

1.2 $$\left({{\text{Y}}}_{1{\text{i}}} - {{\text{Y}}}_{01}\right) \perp {{\text{T}}}_{{\text{i}}} \left|{{\text{X}}}_{{\text{i}}}\right.$$

Assumption (I.2) says that, within a reference class of patients defined by X_i_, treatment choice within the class is *not* influenced by unmeasured patient factors associated with treatment effectiveness or there is no *essential heterogeneity* [38, 39, 45]. If ignorability holds within a reference class defined by X_i_, only the treatment variation that stems from patient factors *unrelated to treatment effectiveness* will be used to estimate treatment effects within the class. Consequently, CFA simulation results which assume ignorability provide no guidance on the properties of patient-specific treatment effect estimates in real-world scenarios in which essential heterogeneity is thought to exist *a priori*. For example, the effectiveness of surgery for patients with shoulder fractures is thought to vary with fracture complexity and patient resiliency, which in turn influence surgery choice [73–77], but fracture complexity and patient resiliency are not measurable in large observational databases such as Medicare claims data [73–77]. A study using a causal forest algorithm to estimate patient-specific surgery effects using Medicare claims data theorized a priori that the resulting estimates should be interpreted in terms of essential heterogeneity, but evidence was not available to guide these interpretations [78]. In addition, understanding influence of essential heterogeneity on CFA estimates is especially relevant to researchers proposing to use CFAs in *effectiveness-implementation hybrid study designs* in which the *promotion* of a treatment is randomized to satisfy assumption (I.1) but decision makers still have the discretion to choose among available treatments based on individual patient factors [79–95].

To provide this guidance, this study modified a treatment choice-based simulation method used in previous research to assess the impact of essential heterogeneity on patient-specific treatment effect estimates from a CFA estimator [43, 48, 53]. Eleven patient populations were simulated with the same distribution of true treatment effects drawn from identical distributions of simulated patient factors. All eleven simulations were specified to satisfy assumption (I.1). The simulations varied by plausible differences in the extent to which knowledge of true patient-specific treatment effects influenced treatment choice. We used the causal forest algorithm within the generalized random forests application (CFA-GRF) [24–26, 96, 97] to estimate patient-specific treatment effects for each simulated population. CFA-GRF has been singled out as the most appropriate CFA for estimating patient-specific treatment effects [98]. To tease out the influence of essential heterogeneity, we applied CFA-GRF to each simulated population under conditions of (1) *fully observed heterogeneity* in which all patient factors associated with treatment effect heterogeneity are observed by the researcher and (2) *partially observed heterogeneity* in which only a subset of the patient factors associated with treatment effect heterogeneity are observed by the researcher. Patient-specific treatment effect estimates from CFA-GRF were used to calculate the average absolute and average percentage differences between true and estimated effects for each simulated population and for treatment choice-based population subsets.

## Methods

### Simulation model

**Fig. 1:**
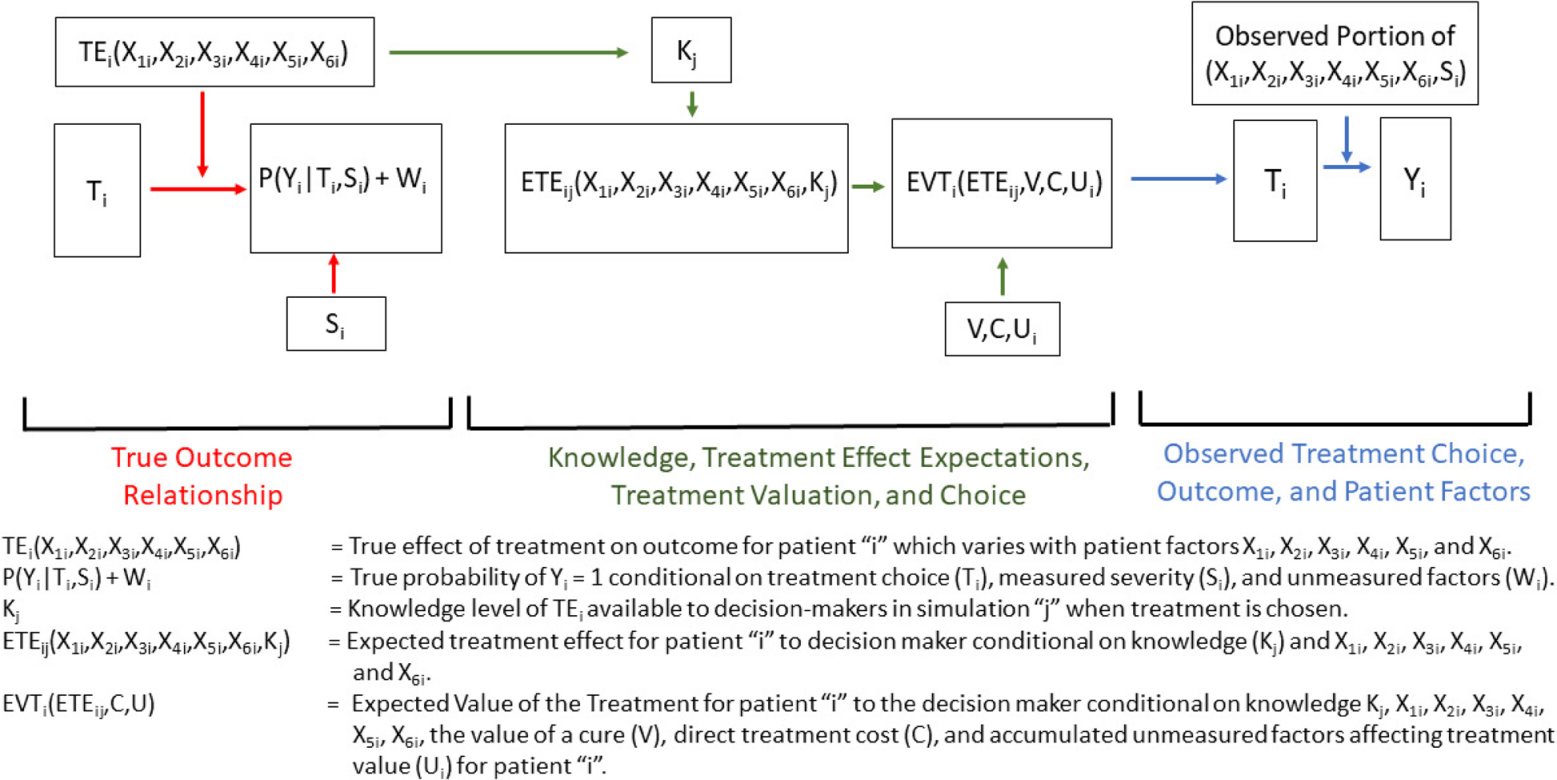
Directed Acyclic Graph (DAG) Describing the Conceptual Framework for the Simulation Model in which Patient Factors Affecting Treatment Effectiveness Affect Treatment Choice through Decision Maker Knowledge

Our simulation model follows the general framework in the essential heterogeneity literature [39, 43, 45, 48, 53, 99]. Figure 1 contains a directed acyclic graph (DAG) illustrating the conceptual framework of treatment effect heterogeneity, treatment choice, and outcome within our simulations. Figure 1 was adapted from standard DAG approaches to reflect patient factors affecting treatment effectiveness and the treatment effect knowledge of the decision maker [100, 101]. Outcome (Y_i_) equals 1 if patient “i” is cured of the medical condition, and 0 if not cured. P(Y_i_|T_i_,S_i_) is the probability of cure for patient “i” conditional on treatment choice (T_i_) and patient severity (S_i_). Patient cure probability also varies with accumulated other factors (W_i_). Treatment (T_i_) equals 1 if the patient receives treatment and 0 otherwise, which we designate as *watchful waiting*. In all simulations, the true absolute treatment effect for each patient “i” (TE_i_) on Y_i_ relative to watchful waiting varies with six factors X_1i_, X_2i_, X_3i_, X_4i_, X_5i_, and X_6i_ based on the following equation:

1 $${{\text{TE}}}_{{\text{i}}}\left({{\text{X}}}_{1{\text{i}},}{{\text{X}}}_{2{\text{i}}}{{\text{X}}}_{3{\text{i}}}{{\text{X}}}_{4{\text{i}}}{{\text{X}}}_{5{\text{i}}}{{\text{X}}}_{6{\text{i}}}\right) = {{\upbeta }_{1}}{*}{{\text{X}}}_{1{\text{i}}} + {{\upbeta }_{2}}{*}{{\text{X}}}_{2{\text{i}}} + {{\upbeta }_{3}}{*}{{\text{X}}}_{3{\text{i}}} + {{\upbeta }_{4}}{*}{{\text{X}}}_{4{\text{i}}} + {{\upbeta }_{5}}{*}{{\text{X}}}_{5{\text{i}}} + {{\upbeta }_{6}}{*}{{\text{X}}}_{6{\text{i}}}$$

**Fig. 2:**
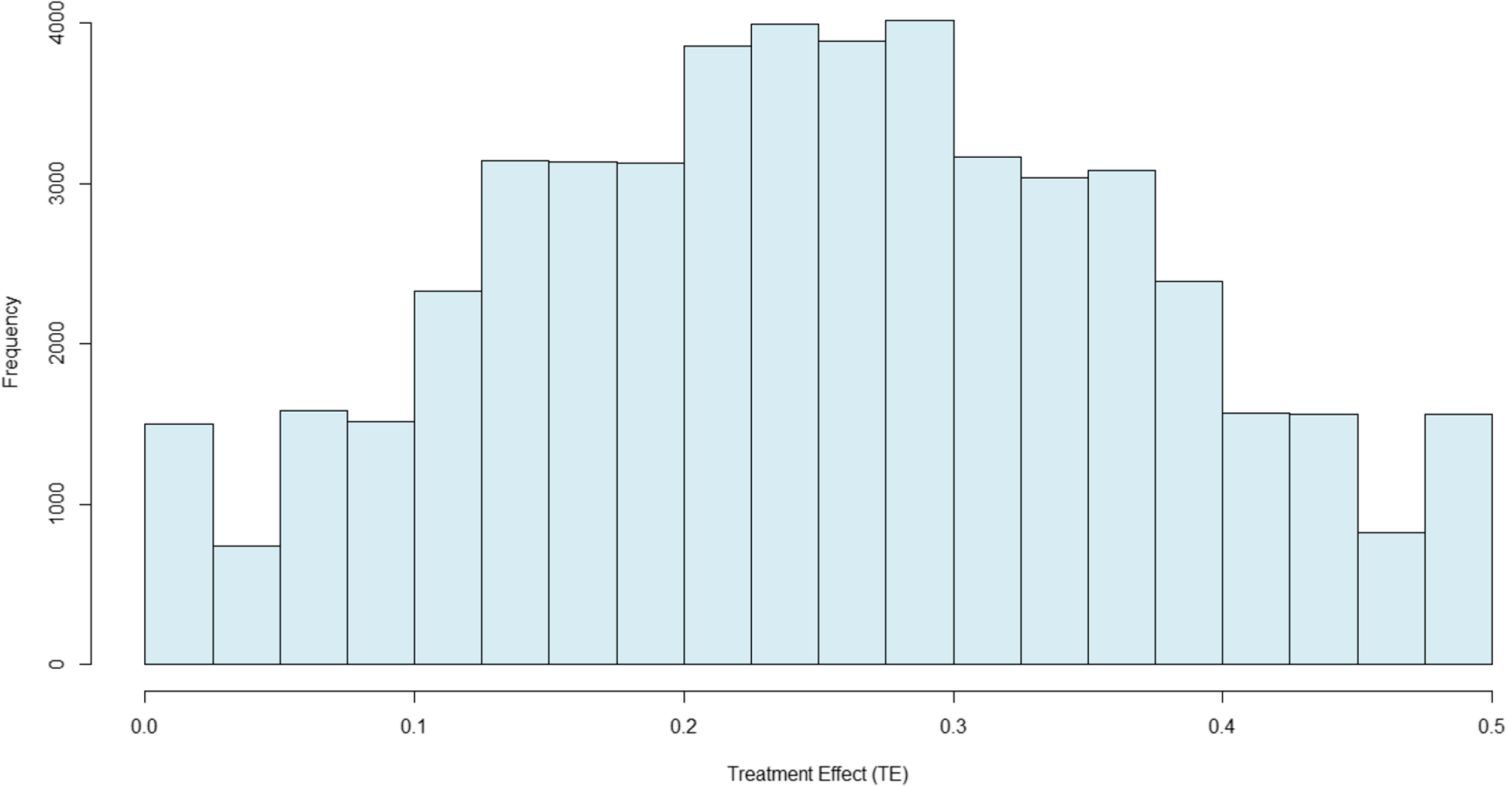
Distribution of True Absolute Treatment Effects (TEi) Used in All Eleven Simulated Populations

X_1i_, X_2i_, X_3i_, X_4i_, X_5i_, and X_6i_ are binary variables distributed Bernoulli for each patient with a probability of 0.5. Each β_x_ equals the absolute change in treatment effect if a patient has condition “X” (β_1_ = 0.024, β_2_ = 0.048, β_3_ = 0.071, β_4_ = 0.095, β_5_ = 0.119, β_6_ = 0.143). With these parameter values, simulated patients have true treatment effects ranging from 0 to 0.5 with an average true treatment effect of 0.25 for each simulated population. For example, if the simulated patient factors for patient “i” (X_1i_,X_2i_,X_3i_,X_4i_,X_5i_,X_6i_) were (1,0,1,0,1,0), then patient “i’s” true TE_i_ was.214 = (0.024 + 0 + 0.071 + 0 + 0.095 + 0). Figure 2 illustrates the identical distribution of simulated treatment effects across all eleven simulations in this study.

The true cure probability relationship for each simulated patient “i” signified by the red arrows in Fig. 1 is as follows:

2 $$\mathrm{Probability}\;\mathrm{of}\;{\mathrm{Y}}_\mathrm{i}\mathrm{=}\mathrm P\left({\mathrm{Y}}_\mathrm{i}\left|{\mathrm{T}}_\mathrm{i},{\mathrm{S}}_\mathrm{i}\right.\right)+{\mathrm{W}}_\mathrm{i}=\left({\mathrm\alpha}_\mathrm{O}+{\mathrm\alpha}_\mathrm{S}\cdot{\mathrm{S}}_\mathrm{i}+{\mathrm{TE}}_\mathrm{i}\left({\mathrm{X}}_{1\mathrm{i}},{\mathrm{X}}_{2\mathrm{i}},{\mathrm{X}}_{3\mathrm{i}},{\mathrm{X}}_{4\mathrm{i}},{\mathrm{X}}_{5\mathrm{i}},{\mathrm{X}}_{6\mathrm{i}}\right)\cdot{\mathrm{T}}_\mathrm{i}\right)+{\mathrm{W}}_\mathrm{i}$$

α_0_ equals the untreated patient cure probability at the mean severity level and was set to 0.1 in all simulations. Patient severity (S_i_) was specified as a uniformly distributed random variable from -0.5 to 0.5. α_S_ equals the change in untreated patient cure probability for differences in severity level and was set to -0.1 in all simulations. As a result, in each simulated population, watchful waiting patients (T_i_ = 0) had a cure probability ranging from 0.05 to 0.15. Treated patients (T_i_ = 1) had a cure probability ranging from 0.05 to 0.65. All other unmeasured patient factors impacting the probability of a cure are found in (W_i_).

The green arrows in Fig. 1 describe the treatment choice process that varied across the eleven simulations. In each simulation, it is assumed that the treatment decision-maker observes X_1i_, X_2i_, X_3i_, X_4i_, X_5i_, and X_6i_ and forms an expected treatment effect for patient “i”. The simulations differ by the *knowledge* available to decision makers of the relationship between the six patient factors and treatment effectiveness, as represented by the expected treatment effect function for simulation “j”:

3 $${\text{ETE}}_\text{ij}\left({\text{X}}_{1\text{i}},{\text{X}}_{2\text{i}},{\text{X}}_{3\text{i}},{\text{X}}_{4\text{i}},{\text{X}}_{5\text{i}},{\text{X}}_{6\text{i}}{\text{K}}_\text{j}\right)={\text{K}}_\text{j}\ast\left({\text{TE}}_\text{i}\left({\text{X}}_{1\text{i}},{\text{X}}_{2\text{i}},{\text{X}}_{3\text{i}},{\text{X}}_{4\text{i}},{\text{X}}_{5\text{i}},{\text{X}}_{6\text{i}}\right)-.25\right)+.25.$$

${{\text{K}}}_{{\text{j}}} \in \left(\text{0, .1, .2, .3, .4, .5, .6, .7, .8, .9, 1}\right)$ is the proportion of patient-specific TE_i_ knowledge used by decision makers in simulation “j” that is distinct from the average population treatment effect. Decision makers are more aware of each patient’s true treatment effect relative to the average population treatment effect as K_j_ increases from 0 to 1 across simulations. For example, in the simulation in which K_j_ = 0, decision makers only have knowledge of the average treatment effect across the population (0.25) when making treatment decisions for each patient. Alternatively, when K_j_ = 1, decision makers have exact knowledge of the treatment effect for patient “i” from observed X_1i_, X_2i_, X_3i_, X_4i_, X_5i_, and X_6i_. ETE_ij_(X_1i_, X_2i_, X_3i_, X_4i_, X_5i_,X_6i_,K_j_) is used to calculate the expected value of treatment for patient “i” based on the following:

4 $${\mathrm{EVT}}_{\mathrm i}\left({\mathrm{ETE}}_{\mathrm{ij}},\mathrm V,\mathrm C,{\mathrm U}_{\mathrm i}\right)=\mathrm V\cdot{\mathrm{ETE}}_{\mathrm{ij}}\left({\mathrm X}_{1\mathrm i},\;{\mathrm X}_{2\mathrm i},\;{\mathrm X}_{3\mathrm i},\;{\mathrm X}_{4\mathrm i},\;{\mathrm X}_{5\mathrm i},\;{\mathrm X}_{6\mathrm i},\;{\mathrm K}_{\mathrm j}\right)-\mathrm C+{\mathrm U}_{\mathrm i}$$

EVT_i_(ETE_ij_,V,C,U_i_) sums the expected benefits and detriments (e.g., costs) of treatment relative to watchful waiting for patient “i” that is conditional on knowledge K_i_, X_1i_,X_2i_,X_3i_,X_4i_,X_5i_,X_6i_, direct treatment cost C, cure value V, and U_i_ other accumulated factors affecting treatment value, which are independent of treatment effectiveness for patient “i”. ETE_ij_(X_1i_, X_2i_, X_3i_, X_4i_, X_5i_,X_6i_,K_j_) equals the decision maker’s expected change in cure probability from treatment. To focus this study on the impact of essential heterogeneity across simulations, all patients were assigned a cure value V of $800 and a treatment cost C of $200. These values were chosen because they yield simulated population treatment percentages of approximately 50%. V designations of $500 and $1100 were also tried, which yielded different population treatment percentages but did not influence the interpretation of our results relative to the essential heterogeneity. U_i_ is the source of treatment valuation that varies across patients, is unrelated to treatment effectiveness and is unmeasured by the researcher. U_i_ values were assigned to patients from a normal distribution with a mean of zero and a common variance $${\sigma }_{U}^{2}$$ across simulations. Furthermore, in all simulations, U_i_ was specified independently of W_i_ so that the differences in unmeasured factors influencing treatment choice had no relationship with the unmeasured factors directly effecting cure so that ignorability assumption (I.1) was satisfied.

In all simulations, decision makers chose treatment for patient “i” if EVT_i_ was positive and watchful waiting if EVT_i_ was negative. In the simulation in which the knowledge of patient-specific treatment effect heterogeneity is zero (K_j_ = 0), only variation in U_i_ leads to different treatment choices across simulated patients. As K_j_ increases across simulations, a larger proportion of the variation in treatment choice variation is attributable to treatment effectiveness or *sorting on the gain*. Once a treatment was chosen for each patient, cure (Y_i_) was simulated using a Bernoulli function of P(Y_i_|T,S_i_) for patient “i”, given T_i_ and S_i_. Table 1 summarizes the model parameters and values used in the simulations.

**Table 1:** Summary of simulation model parameters

Parameter	Description	Value and Distribution
β_1_	Absolute increase in treatment effect on cure when X_1_ = 1	.024
β_2_	Absolute increase in treatment effect on cure when X_2_ = 1	.048
β_3_	Absolute increase in treatment effect on cure when X_3_ = 1	.071
β_4_	Absolute increase in treatment effect on cure when X_4_ = 1	.095
β_5_	Absolute increase in treatment effect on cure when X_5_ = 1	.119
β_6_	Absolute increase in treatment effect on cure when X_6_ = 1	.143
TE_i_	True treatment effect on outcome for patient “i” as a function of X_1i_,X_2i_,X_3i_,X_4i_,X_5i_,X_6i_	Ranges from 0 to .5. Distribution in Fig. 2
S_i_	Patient “i” severity level directly effecting cure but have no effect on treatment effectiveness and are unrelated to treatment choice	Distributed Uniform(-.5,.5)
α_0_	Untreated patient cure probability at mean severity level	.1
α_S_	Change in untreated patient cure probability given S_i_	-.1
V	The value patients gain when cured	$800
C	The cost of treatment	$200
K_j_	The proportion of knowledge of treatment effectiveness that is patient-specific in simulation “j”	$$\left(\begin{array}{c}{0}\text{,} \, \text{.}{1}\text{,} \, \text{.}{2}\text{,} \, \text{.}{3}\text{,} \, \text{.}{4}\text{,} \, \text{.}{5}\text{,} \, \text{.}{6}\text{,} \, \\ \text{.}{7}\text{,} \, \text{.}{8}\text{,} \, \text{.}{9}\text{,} \, {1}\end{array}\right)$$
U_i_	Accumulated unmeasured factors for patient “i” which affect treatment valuation	N(0,25)
ETE_i_	Expected treatment effect for patient “i” given knowledge within simulation “j”	K_j_ * (TE_i_—.25) + .25
EVT_i_	Expected value of treatment for patient “i” given ETE_i_	V• ETE_i_ + C + U_i_
T_i_	1 if patient is EVE_i_ is greater than 1, 0 otherwise	
P(Y_i_|T_i_,S_i_)	Probability patient “i” is cured given TE_i_, T_i_, and S_i_	.1 + TE_i_•T_i_ + (-.1)•S_i_
Y_i_	1 if patient is cured, 0 otherwise	Bernoulli function of P(Y_i_|T_i_,S_i_)
W_i_	Unmeasured patient factors causing variation in Y_i_ given T_i_ and S_i_	

To support large sample properties, we generated 50,000 patients in each simulation. The blue arrows in Fig. 1 describe the variables observed by the researcher after each simulation. By varying the knowledge of TE_i_ across simulations with K_j_ and the patient factors observed by the researcher, we can tease out the impacts of essential heterogeneity on patient-specific treatment effect estimates. In each scenario, researchers observe T_i_, Y_i_, S_i_. We designate “fully observed heterogeneity” as the empirical condition in which researchers observe all six patient factors X_1i_, X_2i_, X_3i_, X_4i_, X_5i_, and X_6i_. We designate “partially observed heterogeneity” as the empirical condition in which researchers observe only X_1i_, X_2i_, X_3i_, and X_4i_. Under fully observed heterogeneity, treatment effects are homogeneous within each reference class spanned by combinations of the complete set of patient factors. When K_j_ = 0, decision-makers are not knowledgeable of the sources of treatment effect heterogeneity, and treatment choice varies only with U_i_. Under fully observed heterogeneity with K_j_ > 0, decision-makers are at least partly knowledgeable of the sources of treatment effect heterogeneity, with the effect of this knowledge on treatment choice increasing with K_j_. Under partially observed heterogeneity, treatment effects are heterogeneous within the reference classes defined by the observed set of patient factors. Partially observed heterogeneity with K_j_ = 0 has been dubbed *nonessential heterogeneity* in the econometric literature [38, 39]. Under nonessential heterogeneity, treatment choice is not influenced by the unmeasured patient factors affecting treatment effectiveness within a reference class. Scenarios with partially observed heterogeneity and K_j_ > 0 represent *essential heterogeneity*. In these scenarios, treatment effects are heterogeneous within each reference class, with the influence of treatment effect heterogeneity on treatment choice increasing with K_j_ across simulations.

### Estimation methods

#### Simulated population summaries

Treatment effect estimation using observational data requires what is called a common area of support or overlap between treated and untreated patients or that patients with the same measured patient factors must be observed to make different treatment choices [102, 103]. It has been shown that including patients in study populations with insufficient overlap can lead to biased treatment effect estimates [104, 105]. The treatment choice-based simulations used here naturally reduce overlap the more that treatment choice is influenced by patient factors affecting treatment effectiveness. To monitor this influence across simulations, we used the SAS PROC LOGISTIC procedure to estimate the treatment propensity score for each patient in each simulated population under both “fully observed heterogeneity” and “partially observed heterogeneity”. Each simulated patient was then designated into either the “overlapped” subset with a propensity score between 0.05 and 0.95 or into the nonoverlapped subset with propensity scores either less than 0.05 or greater than 0.95 [104, 105]. We then estimated the percentage of patients in each simulated population who were treated, untreated, overlapped and treated, overlapped and untreated, nonoverlapped and treated, and nonoverlapped and untreated and then calculated the true average TE_i_ in each subset.

Next, for each simulated population, we estimated a linear probability model (LPM) of treatment choice T_i_ on true TE_i_ using the SAS PROC REG procedure with the SCORR1 option. This procedure provides the percentage of treatment choice variation within the simulated population that is attributable to variation in the true treatment effect to serve as a measure of the influence of the true treatment effect on treatment choice. Last, we estimated the effect of T_i_ and S_i_ on Y_i_ using a LPM in each simulated population. The parametric treatment effect literature states that the LPM estimator of the parameter on T_i_ will yield a consistent estimate of the average absolute treatment effect on the treated in each simulated population [43, 48–50, 54, 57, 60, 68, 69].

#### Casual forest algorithm

We then applied the CFA-GRF [24–26, 96, 97] using the “grf” package in R [106] to estimate treatment effects for each patient in each simulated population. CFA-GRF evolved from standard classification and regression tree (CART) and random forest ensemble methods [24–26, 96, 97]. CART procedures iteratively partition “nodes” of observations within a population into subnodes or “branches” based on measured factors in a manner that maximizes the differences in an outcome across possible branches [97]. A tree is formed by viewing all of the subsequent branches of the study population. The final subnode or leaf on the end of a branch can be thought of as an algorithm-generated *ex post* reference class for observations with factors matching the leaf. The random forest approach is an ensemble method that generates a “forest” of CART trees through resampling from the study population [96]. The estimated outcome for a single observation is the average outcome across the leaves in the trees in the forest containing that observation. CFA-GRF extends the random forest approach to the goal of estimating the causal effect of a predictor of interest (e.g., a treatment) on an outcome. CFA-GRF partitions observations based on measured factors in a manner that maximizes the expected differences *in the estimated treatment effect* on an outcome [24–26]. For each simulated population, CFA-GRF was run using 4000 trees, minimum leaf sizes of 50 and the “honest” approach suggested by the algorithm creators, in which trees were estimated using a randomly selected 25% of the simulated population [26]. We ran CFA-GRF specifying X_1i_, X_2i_, X_3i_, X_4i_, X_5i_, X_6i_, and S_i_ in the “fully observed heterogeneity” specification and X_1i_, X_2i_, X_3i_, X_4i_, and S_i_ in the “partially observed heterogeneity” specification. As a result, each patient in each simulated population had two treatment effect estimates. We assessed the properties of these estimates by evaluating their ability to identify average treatment effect parameters for each simulated population and treatment choice-based subsets of the population. We calculated the average absolute and percentage difference between the true treatment effect for each simulated patient (TE_i_) and estimated treatment effects for the full population and subsets of population based on treatment choice and propensity score “overlap” status.

## Results

### Summary information across simulated populations

Table 2 summarizes each simulated population. Column A in Table 2 shows the proportion of treatment effect expectations (ETE_i_) shaped by the true effect for each patient (TE_i_) in each simulation – K_j_ from Eq. (3). Column B shows the percentage of treatment choice variation in each simulation explained by TE_i_. Columns C and D show the percentage of simulated patients who *overlapped* or had propensity scores greater than 0.05 and less than 0.95 in the fully observed heterogeneity and partially observed heterogeneity scenarios, respectively. Columns E through J show the true average TE_i_ for subsets of treated, untreated, overlapped and treated, overlapped and untreated, nonoverlapped and treated, and nonoverlapped and untreated patients, respectively. These columns also show in parentheses the percentage of patients within each subset.

**Table 2:** Summary information for simulated populations

Simulation	A	B	C	D	E	F	G	H	I	J	K
	Proportion of true (TE_i_) influencing expect treatment effect (ETE_i_) – (K_i_)^a^	% of Treatment Choice Variation Explained by (TE_i_)^b^	Fully Observed Heterogeneity: % of Patients Overlapped^c^	Partially Observed Heterogeneity: % of Patients Overlapped^d^	Average True Absolute Treatment Effect (TE_i_) Within Subset (Percentage of Patients)						Parametric Linear Probability Model Estimate (ATT)
					Full Population		Overlapped with Fully Observed Heterogeneity		Non-Overlapped with Fully Observed Heterogeneity		
					Treated	Untreated	Treated	Untreated	Treated	Untreated	
1	0	.0006	100	100	.250 (49.8)	.251 (50.2)	.250 (49.8)	.251 (50.2)			.249
2	.10	.18	100	100	.256 (49.8)	.246 (50.2)	.256 (49.8)	.246 (50.2)			.255
3	.20	1.4	100	100	.264 (50.0)	.237 (50.0)	.264 (50.0)	.237 (50.0)			.267
4	.30	5.3	100	100	.277 (50.1)	.224 (49.9)	.277 (50.1)	.224 (49.9)			.276
5	.40	11.9	100	100	.290 (50.1)	.212 (49.9)	.290 (50.1)	.212 (49.9)			.292
6	.50	20.1	100	100	.301 (50.2)	.200 (49.8)	.301 (50.2)	.200 (49.8)			.300
7	.60	27.8	97.0	100	.310 (50.2)	.191 (49.8)	.304 (48.7)	.196 (48.3)	.500 (1.5)	.001 (1.5)	.307
8	.70	34.5	90.7	100	.317 (50.3)	.184 (49.7)	.300 (45.5)	.200 (45.2)	.475 (4.8)	.025 (4.5)	.316
9	.80	39.8	84.5	100	.322 (50.2)	.179 (49.8)	.297 (42.3)	.203 (42.1)	.457 (7.9)	.044 (7.6)	.321
10	.90	44.3	78.3	100	.326 (50.2)	.175 (49.8)	.293 (39.2)	.207 (39.1)	.442 (11.0)	.059 (10.7)	.321
11	1.00	48.0	68.8	100	.329 (50.3)	.172 (49.7)	.285 (34.4)	.214 (34.4)	.423 (15.8)	.077 (15.3)	.329

^a^The proportion of patient-specific TE_i_ knowledge used by decision makers in simulation “j” in developing the expected treatment effect for patient “i” that is distinct from the population average treatment effect based on the equation ETE_i_ = K_j_ * (TE_i_(X_1i_,X_2i_,X_3i_,X_4i_,X_5i_,X_6i_)—.25) + .25. The population average treatment effect is .25 in all simulations

^b^The percentage of treatment choice variation explained by TE_i_ using a linear probability model of treatment choice T_i_ on true TE_i_ using SAS PROC REG procedure with the SCORR1 option

^c^Percentage of patients in sample with treatment propensity score greater than .05 and less than .95 when all six patient factors are fully specified in the propensity score equation

^d^Percentage of patients in sample with treatment propensity score greater than .05 and less than .95 when only X_1i_, X_2i_, X_3i_, X_4i_ factors are specified in the propensity score equation

Patient-specific treatment effects (TE_i_) do not influence treatment choice in simulation 1, and as a result, the average true TE_i_ is close to the true population average treatment effect of 0.25 for both treated and untreated patients. Moving from simulations 2 through 11, though, the knowledge of TE_i_ increases in decision making, and TE_i_ explains a larger portion of the variation in treatment choice (column B). Under fully observed heterogeneity, all patients are fully overlapped in simulations 1 through 6. The percentage of overlapping patients falls from 97.0% to 68.8% in simulations 7 through 11. Under the partially observed heterogeneity, all patients overlapped across all simulations. Columns E and F show how the greater influence of TE_i_ on treatment choice leads to sorting on the gain. The average TE_i_ for the treated patients in Column E increased from 0.250 to 0.329 as K increased from 0 to 1, while the average TE_i_ for the untreated patients in Column F fell from 0.251 to 0.172 across this range. Columns G through J stratify treated and untreated patients by overlap status under fully observed heterogeneity. The average TE_i_ of nonoverlapped treated patients (column I) is greater than that of overlapped treated patients (column G). Likewise, the average TE_i_ of nonoverlapping untreated patients (column J) is less than that of overlapping untreated patients (column H). Column K of Table 2 shows the estimated treatment effect for the full population in each simulation using a linear probability model (LPM). A comparison of these estimates with column E confirms that LPM yields estimates of the average treatment effect on the treated (ATT) [57]. When treatment effects are heterogeneous, LPM estimates appropriately generalize to untreated patients only when TE_i_ does not influence treatment choice, as in simulation 1 [57].

### CFA-GRF results under fully observed heterogeneity

Table 3 contains the average percentage differences between the true treatment effects and individual treatment effect estimates from CFA-GRF for each of the eleven simulated populations under fully observed heterogeneity. Estimates are reported for the full population in each simulation and treatment-choice-based subsets. Table A.1 in the Additional file 1 shows these results in terms of average absolute differences between the true treatment effect values and estimated treatment effects. The percentage differences in Table 3 were calculated using the average true treatment effect for each population subset found in Table 2 and the average absolute differences for each subset in Table A.1. For example, the average percentage difference between the estimated and true treatment effect values for the full population in simulation 1 under fully observed heterogeneity is 100*(-0.0014)/0.25 = -0.56%. Column E of Table 3 shows that under fully observed heterogeneity on average, CFA-GRF produces treatment effect estimates that reflect each population across simulations. However, as treatment choice becomes more responsive to TE_i_, CFA-GRF estimates increasingly understate the true treatment effect for treated patients and overstate the true treatment effect for untreated patients. Simulation 1 under fully observed heterogeneity fully satisfies ignorability, and CFA-GRF produces patient-specific treatment effect estimates that on average reflect the true patient treatment effects for the entire population and for both treated and untreated patient subsets. In contrast, in simulation 11, in which decision-makers have full knowledge of TE_i_ in treatment choice, the treatment effect estimates for treated patients are on average 14.74% lower than the truth, and the estimated treatment effects for untreated patients are on average 30.99% higher than the truth. These percentage differences are not symmetric because untreated patients have a lower average true treatment effect. Columns G to J in simulations 6 through 11 demonstrate that these differences exist for both overlapping and nonoverlapping patients but are more pronounced for nonoverlapping patients.

**Table 3:** Average Percentage Differences Between the Estimated Treatment Effects and True Treatment Effects from the Causal Forest Algorithm within the Generalized Random Forests Application (CFA-GRF) Under *Fully Observed Heterogeneity* Across Simulated Populations Which Differ by the Extent That Treatment Effect Influences Treatment Choice

	A	B	C	D	E	F	G	H	I	J
Simulation	Proportion of true (TE_i_) influencing (ETE_i_) at Treatment Choice – (K_j_)^a^	% of Treatment Choice Variation Explained by (TE_i_)^b^	% of Patients Overlapped^c^	Average Percentage Difference Between True and Estimated Treatment Effects						
				Full Population	Treated	Untreated	Overlapped with Fully Observed Heterogeneity		Non-Overlapped with Fully Observed Heterogeneity	
							Treated	Untreated	Treated	Untreated
1	0	.0006	100	-0.56%	-0.64%	-0.44%				
2	.10	.18	100	-0.36%	-0.39%	-0.28%				
3	.20	1.4	100	0.56%	0.38%	0.76%				
4	.30	5.3	100	-0.84%	-1.12%	-0.54%				
5	.40	11.9	100	0.48%	-1.21%	2.83%				
6	.50	20.1	100	-0.24%	-3.55%	4.75%				
7	.60	27.8	97.0	2.76%	-1.84%	10.31%	-1.15%	8.62%	-14.98%	11110.00%
8	.70	34.5	90.7	-0.96%	-9.78%	14.51%	-7.07%	8.35%	-26.27%	508.00%
9	.80	39.8	84.5	2.84%	-7.74%	22.07%	-3.13%	11.48%	-23.85%	293.41%
10	.90	44.3	78.3	0.08%	-11.66%	22.11%	-5.53%	8.79%	-26.11%	192.88%
11	1.00	48.0	68.8	0.84%	-14.74%	30.99%	-5.33%	10.47%	-28.58%	159.22%

^a^The proportion of patient-specific TE_i_ knowledge used by decision makers in simulation “j” in developing the expected treatment effect for patient “i” that is distinct from the population average treatment effect based on the equation ETE_i_ = K_j_ * (TE_i_(X_1i_,X_2i_,X_3i_,X_4i_,X_5i_,X_6i_)—.25) + .25

^b^The percentage of treatment choice variation explained by TE_i_ using a linear probability model of treatment choice T_i_ on true TE_i_ using SAS PROC REG procedure with the SCORR1 option

^c^Percentage of patients in sample with treatment propensity score greater than .05 and less than .95 when all six patient factors are fully specified in the propensity score equation

### CFA-GRF results under partially observed heterogeneity

Table 4 contains the average percentage differences between the true treatment effect values and CFA-GRF treatment effect estimates for each simulated population under partially observed heterogeneity. Under partially observed heterogeneity all patients are overlapped so that the columns G through J found in Table 3 are unnecessary. Under ignorability in simulation 1, CFA-GRF again produces estimates that on average are close to true patient treatment effects for the entire population and for the treated and untreated patient subsets. In simulation 1, CFA-GRF estimates under partially observed heterogeneity had larger standard errors than those under fully observed heterogeneity (see Table A.2). Treatment effects estimated from CFA-GRF for treated patients closely reflect their true values across all eleven simulations. In contrast, CFA-GRF estimates for untreated patients are higher than their true values across simulations 2 through 11, with the differences increasing with the level of TE_i_ influence on treatment choice. For example, based on the true average treatment effect for untreated patients from Table 2 and the average absolute differences for each population in Table A.1, on average, CFA-GRF estimates for untreated patients are 2.4% greater than their true values in simulation 2 – 100*(0.006)/(0.246)) and 76.3% greater than their true values in simulation 11 – 100*(0.1312)/(0.172). As a result, when TE_i_ influences treatment choice under partially observed heterogeneity, CFA-GRF estimated treatment effects across the whole population are on average greater than their true values.

**Table 4:** Average Percentage Differences Between the Estimated Treatment Effects and True Treatment Effects from the Causal Forest Algorithm within the Generalized Random Forests Application (CFA-GRF) Under *Partially Observed Heterogeneity* Across Simulated Populations Which Differ by the Extent That Treatment Effect Influences Treatment Choice

Simulation	A	B	C	D	E	F
	Proportion of true (TE_i_) influencing (ETE_i_) at Treatment Choice – (K_j_)^a^	% of Treatment Choice Variation Explained by (TE_i_)^b^	% of Patients Overlapped^c^	Average Percentage Difference Between True and Estimated Treatment Effects		
				Full Population	Treated	Untreated
1	0	.0006	100	-1.16%	-1.08%	-1.27%
2	.10	.18	100	1.12%	-0.20%	2.44%
3	.20	1.4	100	4.12%	0.57%	8.02%
4	.30	5.3	100	6.44%	-0.36%	14.87%
5	.40	11.9	100	12.12%	0.72%	27.69%
6	.50	20.1	100	15.08%	0.27%	37.40%
7	.60	27.8	100	18.68%	0.58%	48.22%
8	.70	34.5	100	18.60%	-1.51%	53.42%
9	.80	39.8	100	24.36%	1.27%	66.03%
10	.90	44.3	100	22.00%	-1.75%	66.51%
11	1.00	48.0	100	25.44%	-1.03%	76.28%

^a^The proportion of patient-specific TE_i_ knowledge used by decision makers in simulation “j” in developing the expected treatment effect for patient “i” that is distinct from the population average treatment effect based on the equation ETE_i_ = K_j_ * (TE_i_(X_1i_,X_2i_,X_3i_,X_4i_,X_5i_,X_6i_)—.25) + .25

^b^The percentage of treatment choice variation explained by TE_i_ using a linear probability model of treatment choice T_i_ on true TE_i_ using SAS PROC REG procedure with the SCORR1 option

^c^Percentage of patients in sample with treatment propensity score greater than .05 and less than .95 when only X_1i_, X_2i_, X_3i_, X_4i_ factors are specified in the propensity score equation

## Discussion

Causal forest algorithms (CFAs) have been proposed to estimate patient-specific treatment effect evidence using observational data [23–33, 107]. To apply CFAs, observational databases must contain patients with similar combinations of measured factors who were observed to make different treatment choices. The positive properties of CFAs for estimating patient-specific treatment effects have been established using simulation models under the assumption of ignorability [26–29, 34–36]. Under ignorability, only the treatment variation from *unobserved patient factors not associated with treatment effect heterogeneity* is available to estimate patient-specific treatment effects. Therefore, it is unknown whether the positive properties of CFAs extend to real-world clinical applications in which patient factors affecting treatment effectiveness also influence treatment choice. In many real-world clinical scenarios it is plausible and likely that observed treatment choices reflect unmeasured patient factors related to expected treatment effectiveness for each patient – a condition defined in econometric literature as *essential heterogeneity* [38, 39, 43, 48–50, 53]. This paper used simulations that varied only by the relationship between treatment effectiveness and treatment choice to assess the impact of essential heterogeneity on the ability of CFAs to estimate patient-specific treatment effects. The causal forest algorithm within the generalized random forests application CFA-GRF has been singled out as most appropriate CFA estimate patient-specific treatment effects and was used here [98]. To tease out the impacts of essential heterogeneity, CFA-GRF estimates were evaluated in settings in which all patient factors associated with treatment effect heterogeneity were fully observed by the researcher and in settings in which the patient factors associated with treatment effect heterogeneity were not fully observed by the researcher.

We replicated the positive properties of CFA-GRF in simulation scenarios under ignorability. CFA-GRF yielded average population-wide estimates and average estimates by patient subsets based on treatment choice under ignorability that were closely aligned with their true values whether heterogeneity was fully or partially observed within the algorithm. As a result, if researchers can make a strong conceptual case a priori that treatment effectiveness is unrelated to treatment choice, they can be confident that CFA-GRF can yield appropriate treatment effect estimates across a population of patients. In simulation scenarios in which decision-makers use patient factors associated with treatment effectiveness in making treatment decisions [38, 39, 43, 48–50, 53], the ability of CFA-GRF to identify patient-specific treatment effects varied with the influence that treatment effectiveness had on treatment choice and whether the full range of patient factors associated with treatment effect heterogeneity were observed and specified in the algorithm. When all patient factors affecting treatment effect heterogeneity were fully specified, CFA-GRF produced treatment effect estimates that reflected true treatment effects across each population subset when the influence of treatment effectiveness on treatment choice was low. As this influence increased, however, treatment effect estimates showed increasingly negative bias for treated patients and positive bias for untreated patients. A substantial portion of this bias is likely attributable to nonoverlapping patients becoming a higher percentage of patients as the influence of treatment effectiveness on treatment choice increases. Under partially observed heterogeneity, all patients overlapped in all simulations. CFA-GRF produced estimates that closely reflected the true treatment effect values for treated patients across all levels of influence of treatment effectiveness on treatment choice. In contrast, CFA-GRF estimates for untreated patients were biased high, with the extent of this bias increasing with the level of influence that treatment effectiveness had on treatment choice.

As a result, CFA-GRF estimates of patient-specific treatment effects using observational data must be assessed through the prism of the assumed reasons why patients with similar measured factors in a real-world setting were observed making different treatment choices. This requires researchers to explicitly develop conceptual frameworks of treatment choice to support these assumptions a priori to ensure proper interpretation of treatment effect estimates *ex post*. The call for treatment choice conceptual frameworks to guide treatment effectiveness research using observational data has long been stated in economics [44, 48, 49, 108–110], and the importance of these frameworks is now being more widely appreciated [21, 111, 112]. A conceptual framework of treatment choice should describe the factors thought to influence treatment choice, the relationship of these factors to treatment effectiveness and whether these factors are measured within the available data. Given the study findings, it would be important for researchers to qualify patient-specific estimates from CFA-GRF in clinical scenarios in which essential heterogeneity likely exists. In these scenarios researchers should state that patient-specific estimates from CFA-GRF are likely biased high for the average patient with a given combination measured patient factors and are best aligned to those patients a provider is more likely to treat.

This study is limited by its use of only using one of the several CFAs available to produce patient-specific evidence using observational data. While the CFA-GRF was singled out as most appropriate for estimating patient-specific treatment effects [98], it is possible that other CFAs are available that can incorporate and correct for the conditions associated with treatment choice when making treatment effect estimates. To this end, the simulated datasets produced here are available from the authors for use by other CFA developers to assess the impact on treatment effect estimates of the influence of treatment effect heterogeneity on treatment choice. In addition, the simulation approach in this paper is reported fully, is straightforward to reproduce, and is easy to modify, so researchers can assess the robustness of our results to parameter changes.

## Conclusion

The acknowledged breadth of *treatment effect heterogeneity* across patients heightens the need to find empirical approaches to find patient-specific treatment effect evidence [4–10]. Causal forest algorithms (CFAs) have been proposed to analyze the treatment variation found within large observational databases to develop patient-specific evidence [23–33]. The simulation results in this paper show that the patient-specific estimates produced by a CFA are sensitive to the reasons why patients with the same set of measured factors were observed to make different treatment choices. It is likely in many real-world clinical scenarios that decision-makers are cognizant of how patient factors affect treatment effectiveness and use this information in making treatment decisions [38, 39, 43, 48–50, 53]. And many real-world decision makers may know more about the list of patient factors affecting treatment effectiveness than the researchers who collect measures for research [22, 113, 114]. As a result, it is foundational that researchers using CFAs to estimate patient-specific evidence using observational data build conceptual frameworks of treatment choice prior to estimation to guide estimate interpretation ex post.

### Supplementary Information

**Supplementary Material 1.**

## Data Availability

No datasets were generated or analysed during the current study.

## Abbreviations

CFA: Causal forest algorithm
ATT: Average treatment effect on the treated
DAG: Directed acyclic graph
CART: Classification and regression tree
CFA-GRF: Causal forest algorithm - generalized random forest application
