# Supplementary material for: Assessing the properties of patient-specific treatment effect estimates from causal forest algorithms under essential heterogeneity
Source: BMC Med Res Methodol. 2024 Mar 13;24:66. doi: 10.1186/s12874-024-02187-5 (PMC10935905; doi:10.1186/s12874-024-02187-5)
Supplement: Supplementary file 1 — Supplementary Material 1. [file 12874_2024_2187_MOESM1_ESM.docx]

| Table A.1: Average Absolute Differences Between the Estimated Treatment Effects and True Treatment Effects from the Causal Forest Algorithm within the Generalized Random Forests Application (CFA-GRF) Across Simulated Populations Which Differ by the Extent That Treatment Effect Influences Treatment Choice. | | | | | | | | | | | |
| --- | --- | --- | --- | --- | --- | --- | --- | --- | --- | --- | --- |
|  | A | B | C | D | E | F | G | H | I | J | K |
| Simulation | Proportion of true (TE_i_) influencing (ETE_i_) at Treatment Choice -- (K_i_)^a^ | % of Treatment Choice Variation Explained by (TE_i_)^b^ | Fully Observed Heterogeneity:  % of Patients Overlapped^c^ | Partially Observed Heterogeneity:  % of Patients Overlapped^d^ | Average Absolute Difference Between True and Estimated Treatment Effects | | | | | | |
|  |  |  |  |  | Full Population | Treated | Untreated | Overlapped with Fully Observed Heterogeneity | | Non-Overlapped with Fully Observed Heterogeneity | |
|  |  |  |  |  |  |  |  | Treated | Untreated | Treated | Untreated |
| Fully Observed Heterogeneity | | | | | | | | | | | |
| 1 | 0 | .0006 | 100 |  | -.0014 | -.0016 | -.0012 |  |  |  |  |
| 2 | 10 | .18 | 100 |  | -.0009 | -.0010 | -.0007 |  |  |  |  |
| 3 | 20 | 1.4 | 100 |  | .0014 | .0010 | .0018 |  |  |  |  |
| 4 | 30 | 5.3 | 100 |  | -.0021 | -.0031 | -.0012 |  |  |  |  |
| 5 | 40 | 11.9 | 100 |  | .0012 | -.0035 | .0060 |  |  |  |  |
| 6 | 50 | 20.1 | 100 |  | -.0006 | -.0107 | .0095 |  |  |  |  |
| 7 | 60 | 27.8 | 97.0 |  | .0069 | -.0057 | .0197 | -.0035 | .0169 | -.0749 | .1111 |
| 8 | 70 | 34.5 | 90.7 |  | -.0024 | -.0310 | .0267 | -.0212 | .0167 | -.1248 | .1270 |
| 9 | 80 | 39.8 | 84.5 |  | .0071 | -.0250 | .0395 | -.0093 | .0233 | -.1090 | .1291 |
| 10 | 90 | 44.3 | 78.3 |  | .0002 | -.0380 | .0387 | -.0162 | .0182 | -.1154 | .1138 |
| 11 | 100 | 48.0 | 68.8 |  | .0021 | -.0485 | .0533 | -.0152 | .0224 | -.1209 | .1226 |
| Partially Unobserved Heterogeneity | | | | | | | | | | | |
| 1 | 0 | .0006 |  | 100 | -.0029 | -.0027 | -.0032 |  |  |  |  |
| 2 | 10 | .18 |  | 100 | .0028 | -.0005 | .0060 |  |  |  |  |
| 3 | 20 | 1.4 |  | 100 | .0103 | .0015 | .0190 |  |  |  |  |
| 4 | 30 | 5.3 |  | 100 | .0161 | -.0010 | .0333 |  |  |  |  |
| 5 | 40 | 11.9 |  | 100 | .0303 | .0021 | .0587 |  |  |  |  |
| 6 | 50 | 20.1 |  | 100 | .0377 | .0008 | .0748 |  |  |  |  |
| 7 | 60 | 27.8 |  | 100 | .0467 | .0018 | .0921 |  |  |  |  |
| 8 | 70 | 34.5 |  | 100 | .0465 | -.0048 | .0983 |  |  |  |  |
| 9 | 80 | 39.8 |  | 100 | .0609 | .0041 | .1182 |  |  |  |  |
| 10 | 90 | 44.3 |  | 100 | .0550 | -.0057 | .1164 |  |  |  |  |
| 11 | 100 | 48.0 |  | 100 | .0636 | -.0034 | .1312 |  |  |  |  |
| a. The proportion of patient-specific TE_i_ knowledge used by decision makers in simulation “j” in developing the expected treatment effect for patient “i” that is distinct from the population average treatment effect based on the equation ETE_i_ = K_j_ * (TE_i_(X_1i_,X_2i_,X_3i_,X_4i_,X_5i_,X_6i_) - .25) + .25.  b. The percentage of treatment choice variation explained by TE_i_ using a linear probability model of treatment choice T_i_ on true TE_i_ using SAS PROC REG procedure with the SCORR1 option.  c. Percentage of patients in sample with treatment propensity score greater than .05 and less than .95 when all six patient factors are fully specified in the propensity score equation.  d. Percentage of patients in sample with treatment propensity score greater than .05 and less than .95 when only X_1i_, X_2i_, X_3i_, X_4i_ factors are specified in the propensity score equation. | | | | | | | | | | | |

| Table A.2: Standard Errors of the Average Absolute Differences Between the Estimated Treatment Effects and True Treatment Effects from the Causal Forest Algorithm within the Generalized Random Forests Application (CFA-GRF) Across Simulated Populations Which Differ by the Extent That Treatment Effect Influences Treatment Choice. | | | | | | | | | | | |
| --- | --- | --- | --- | --- | --- | --- | --- | --- | --- | --- | --- |
|  | A | B | C | D | E | F | G | H | I | J | K |
| Simulation | Proportion of true (TE_i_) influencing (ETE_i_) at Treatment Choice -- (K_i_)^a^ | % of Treatment Choice Variation Explained by (TE_i_)^b^ | Fully Observed Heterogeneity:  % of Patients Overlapped^c^ | Partially Observed Heterogeneity:  % of Patients Overlapped^d^ | Average Absolute Difference Between True and Estimated Treatment Effects | | | | | | |
|  |  |  |  |  | Full Population | Treated | Untreated | Overlapped with Fully Observed Heterogeneity | | Non-Overlapped with Fully Observed Heterogeneity | |
|  |  |  |  |  |  |  |  | Treated | Untreated | Treated | Untreated |
| Fully Observed Heterogeneity | | | | | | | | | | | |
| 1 | 0 | .0006 | 100 |  | 0.0002 | 0.0003 | 0.0003 |  |  |  |  |
| 2 | 10 | .18 | 100 |  | 0.0002 | 0.0003 | 0.0003 |  |  |  |  |
| 3 | 20 | 1.4 | 100 |  | 0.0002 | 0.0003 | 0.0003 |  |  |  |  |
| 4 | 30 | 5.3 | 100 |  | 0.0002 | 0.0003 | 0.0003 |  |  |  |  |
| 5 | 40 | 11.9 | 100 |  | 0.0002 | 0.0003 | 0.0003 |  |  |  |  |
| 6 | 50 | 20.1 | 100 |  | 0.0002 | 0.0003 | 0.0003 |  |  |  |  |
| 7 | 60 | 27.8 | 97.0 |  | 0.0003 | 0.0003 | 0.0003 | .0003 | .0003 | .0017 | .0017 |
| 8 | 70 | 34.5 | 90.7 |  | 0.0003 | 0.0004 | 0.0004 | .0003 | .0003 | .0011 | .0011 |
| 9 | 80 | 39.8 | 84.5 |  | 0.0004 | 0.0004 | 0.0004 | .0004 | .0004 | .0009 | .0009 |
| 10 | 90 | 44.3 | 78.3 |  | 0.0005 | 0.0005 | 0.0005 | .0005 | .0005 | .0009 | .0010 |
| 11 | 100 | 48.0 | 68.8 |  | 0.0004 | 0.0005 | 0.0005 | .0004 | .0004 | .0006 | .0006 |
| Partially Unobserved Heterogeneity | | | | | | | | | | | |
| 1 | 0 | .0006 |  | 100 | 0.0005 | 0.0006 | 0.0006 |  |  |  |  |
| 2 | 10 | .18 |  | 100 | 0.0005 | 0.0006 | 0.0006 |  |  |  |  |
| 3 | 20 | 1.4 |  | 100 | 0.0005 | 0.0006 | 0.0006 |  |  |  |  |
| 4 | 30 | 5.3 |  | 100 | 0.0004 | 0.0006 | 0.0006 |  |  |  |  |
| 5 | 40 | 11.9 |  | 100 | 0.0005 | 0.0006 | 0.0006 |  |  |  |  |
| 6 | 50 | 20.1 |  | 100 | 0.0005 | 0.0006 | 0.0006 |  |  |  |  |
| 7 | 60 | 27.8 |  | 100 | 0.0005 | 0.0006 | 0.0006 |  |  |  |  |
| 8 | 70 | 34.5 |  | 100 | 0.0005 | 0.0006 | 0.0006 |  |  |  |  |
| 9 | 80 | 39.8 |  | 100 | 0.0005 | 0.0005 | 0.0006 |  |  |  |  |
| 10 | 90 | 44.3 |  | 100 | 0.0005 | 0.0005 | 0.0005 |  |  |  |  |
| 11 | 100 | 48.0 |  | 100 | 0.0005 | 0.0005 | 0.0005 |  |  |  |  |
| a. The proportion of patient-specific TE_i_ knowledge used by decision makers in simulation “j” in developing the expected treatment effect for patient “i” that is distinct from the population average treatment effect based on the equation ETE_i_ = K_j_ * (TE_i_(X_1i_,X_2i_,X_3i_,X_4i_,X_5i_,X_6i_) - .25) + .25.  b. The percentage of treatment choice variation explained by TE_i_ using a linear probability model of treatment choice T_i_ on true TE_i_ using SAS PROC REG procedure with the SCORR1 option.  c. Percentage of patients in sample with treatment propensity score greater than .05 and less than .95 when all six patient factors are fully specified in the propensity score equation.  d. Percentage of patients in sample with treatment propensity score greater than .05 and less than .95 when only X_1i_, X_2i_, X_3i_, X_4i_ factors are specified in the propensity score equation. | | | | | | | | | | | |
